# Undetectable = Untransmittable (U = U) Awareness and HIV Treatment Optimism Among Men Who Have Sex with Men in Mumbai, India

**DOI:** 10.1007/s10461-026-05063-z

**Published:** 2026-02-24

**Authors:** Jatin Chaudary, Shruta Rawat, Alpana Dange, Sarit A. Golub, Ryung S. Kim, Naveen Mutyala, Venkatesan Chakrapani, Kenneth H. Mayer, Julia Arnsten, Viraj V. Patel

**Affiliations:** 1 Research Unit, The Humsafar Trust, Mumbai, India; 2 University of Toronto India Foundation, Mumbai, India; 3 Independent Research Consultant, Mumbai, India; 4 Department of Psychology, Hunter College, City University of New York, New York, NY USA; 5 Department of Epidemiology and Population Health, Albert Einstein College of Medicine, Bronx, NY USA; 6 Centre for Sexuality and Health Research and Policy, Chennai, India; 7 The Fenway Institute, Fenway Health, Boston, MA USA; 8 Department of Medicine, Beth Israel Deaconess Medical Center, Harvard Medical School, Boston, MA USA; 9 Division of General Internal Medicine, Department of Medicine, Albert Einstein College of Medicine, Montefiore Health System, 1300 Morris Park Avenue, Bronx, NY 10461 USA

**Keywords:** Treatment as prevention, Viral suppression, Stigma, Antiretroviral therapy, HIV Infections/Prevention & control

## Abstract

Little is known about Undetectable=Untransmittable (U = U) awareness among MSM in India. We analyzed baseline survey data from MSM (*N* = 1004) in an mHealth HIV testing trial in Mumbai and Thane, India. We assessed factors associated with U = U awareness and agreement (HIV treatment optimism) using multivariable Poisson regression. U = U awareness (29%) was associated with English survey completion, searching for sexual health information online, awareness of PrEP/PEP, and participation in MSM-focused HIV prevention programs (all *P* < 0.05). Overall, 50% of the participants agreed with HIV treatment optimism, which was associated with searching for sexual health information online, PrEP knowledge, and being out about their sexual orientation. Focused, culturally tailored U = U education is needed to improve awareness and treatment optimism among diverse Indian MSM.

**Supplementary Information:**

The online version contains supplementary material available at 10.1007/s10461-026-05063-z.

## Introduction

The HIV epidemic remains a pressing public health concern globally, with India having the third highest HIV burden in the world. In 2023, India reported 2.54 million people living with HIV (PLHIV) at an estimated adult HIV prevalence of 0.20% [1]. Notably, HIV sentinel surveillance data find that the epidemic remains concentrated within key populations, particularly men who have sex with men (MSM), who had a prevalence rate of 3.26% in 2023, much higher than the general population [1]. Targeted Interventions (TIs), initiated under the National AIDS Control Programme, provide a comprehensive package of prevention, care, support, and treatment services to communities at high risk, including MSM. However, despite these efforts, HIV prevalence among MSM in India remains disproportionately high. With an aim to control the elevated HIV burden and facilitate early linkage of newly diagnosed individuals, the Government of India in April 2017, introduced the ‘Test and Treat Policy for HIV,’ ensuring that all PLHIV receive antiretroviral therapy (ART) immediately, regardless of CD4 count, clinical stage, age or population group [2].

The concept Undetectable = Untransmittable (U = U) indicates that individuals living with HIV who achieve and maintain an undetectable viral load through consistent ART adherence cannot transmit HIV to their sexual partners even without other preventive methods [3]. U = U could help reduce stigma and fear, empower affected communities, encourage HIV testing and treatment, and support public health goals. Studies show that U = U awareness is associated with reduced HIV-related misconceptions and stigma, improved sexual health behaviors, and expansion in HIV testing and prevention strategies [3, 4]. HIV treatment optimism, which reflects belief in the effectiveness of HIV treatment, has been linked to HIV incidence and behaviors associated with increased HIV vulnerability, such as condomless sex [5]. However, this has not been explored in depth among HIV-negative MSM in India.

While the majority of studies on U = U awareness and HIV treatment optimism originate from high-income settings [6], awareness of U = U and HIV treatment optimism among MSM in India remains largely unexplored, with only one study assessing U = U awareness among a sample of Indian sexual and gender minorities recruited from one dating app, finding that only 14% were aware of U = U [7]. We thus sought to explore U = U awareness and HIV treatment optimism among MSM reached online and identify associated factors.

## Methods

### Study Design, Participants, and Data Collection

We conducted a secondary cross-sectional analysis using baseline data from participants enrolled in an mHealth-based parallel randomized control trial: *Chalo! 2.0*, in Mumbai and Thane, India. The trial’s primary aim was to increase HIV testing uptake among sexually active MSM, as described elsewhere [8]. Briefly, the parent study provided all participants with a digital coupon for free HIV testing options (at an LGBT CBO, self-testing, or at a private lab), randomized participants to an HIV-focused educational arm, a general health promotion arm, or a digital coupon-only arm and followed participants for up to 18-months.We recruited participants from Internet-based platforms (e.g., social media, dating apps, WhatsApp) using multiple strategies, including paid advertisements and virtual peer-to-peer outreach.

Potential participants clicked on a study link directed to an eligibility screener. Eligible participants provided online consent, had to verify their WhatsApp number, and then received a baseline survey link on WhatsApp. The baseline survey was a web-based, self-administered survey that could be completed in English or Hindi (per participant choice). As part of the parent study’s eligibility, all participants had to authenticate their WhatsApp number by clicking on a WhatsApp-based link sent on the provided WhatsApp number before being able to initiate the baseline survey. The process was automated at the backend by the study’s technology partner and sent through a verified WhatsApp business account. The survey design incorporated measures to minimize duplicate or repeat submissions. Participants could not submit incomplete surveys, and each registered phone number was permitted to complete the baseline survey only once. Additional checks, such as duplicate online payment IDs (for digital payments as incentives), phone numbers, and IP addresses, were used to identify potential duplicates (reported previously in detail). In addition to questions on U = U awareness and HIV treatment optimism, the survey collected information on demographics, HIV testing behavior, knowledge of PrEP and PEP, social support, and outness, with details being previously published [8]. Participants could skip certain questions; however, questions related to U = U, HIV testing, and behaviors among others were mandatory. Upon successful submission of the survey, they were considered enrolled and compensated INR 300 (USD $3.50).

A goal of the parent study was to engage individuals unaware of their current HIV status in line with India’s National AIDS Control Organization’s recommendation of HIV testing every 6-months for sexually active MSM. Eligibility criteria for the parent study and this analysis included being 18 years or older, living or working in the Mumbai metropolitan area (including Thane) fluent in Hindi or English, male sex at birth, current gender identity as male, having anal sex with men in the past year, and either never tested for HIV, or were unaware of their results, or were reported being HIV-negative and testing > 6 months ago but had anal sex since the last test [8].

### Measures and Statistical Analysis

The dependent variables for this analysis were awareness of U = U and HIV treatment optimism. Awareness was assessed with the question: “Have you heard of the statement Undetectable = Untransmittable (U = U) before today?” Participants who responded “Yes” were classified as aware of U = U, while those who responded “No” were classified as unaware. Prior to the awareness question, information on U = U was provided, stating: *“Undetectable = Untransmittable (U = U) means that a person living with HIV is considered ‘undetectable’ when HIV treatment lowers HIV levels in the body to very low levels that tests cannot detect. As long as the person takes their treatment and remains undetectable*,* they cannot pass HIV to others through sex.”* HIV treatment optimism was measured with agreement to the statement: “People living with HIV who take their treatment regularly and have an undetectable viral load, can have a healthy and long life” and responses dichotomized to “Agree” (strongly agree/agree) and “Disagree” (neither/disagree/strongly disagree). Independent variables included sociodemographic characteristics (age, education, employment, income, and language) and HIV-related factors such as HIV testing history, access to HIV testing, HIV risk perception, awareness of PrEP and PEP, history of sex work, condom use, and participation in Targeted HIV Prevention Intervention (TIs). Other factors included searching for sexual health information online, someone to talk about sex concerns, outness, sexual orientation, and source of enrolment.

Outness was assessed based on the response to the following question, “How ‘OUT’ would you say you are about your sexual identity or attraction to other men?” Respondents were allowed to select multiple responses, including: “Out to no one,” “Out to everyone I know,” “Out to other LGBTQ+ people/MSM,” “Out to some/all immediate family,” “Out to some/all extended family,” and “Out to only some/all heterosexual friends.” Similar responses were grouped, resulting in three categories of outness: (1) out to someone, (2) out to LGBTQ+ individuals, and (3) out to family.

We used summary statistics to describe the sample and then analyzed the outcomes using bivariate analysis (Chi-square). To further examine associations, prevalence ratios (adjusted relative risks) were estimated using Poisson regression (i.e., generalized linear model with log-link), robust standard errors, and a 95% confidence interval. A *p*-value of less than 0.05 was considered significant. Due to high multicollinearity and the large number of variables, Poisson regression models with a least absolute shrinkage and selection operator (LASSO) penalty were applied. LASSO selects a single variable from a group of highly correlated predictors due to the sparsity-inducing property of L1 penalty in which the coefficients of others covariates are often estimated as zero. Fifty-fold cross-validation was performed across 100 values of λ (the penalization parameter) to identify the optimal model, which minimized deviance. This process involved fitting 100 models 50 times for each outcome, with age included as a mandatory variable. Additional details regarding the LASSO modelling procedure and retention of variable are presented in Supplementary Fig. 1. All analyses were conducted using R statistical software.

### Ethics Approval

The IRBs of The Humsafar Trust (HST) (India) (HST-IRB-42/2-01/2022) and Albert Einstein College of Medicine (United States) (2018–9471) approved the study and cleared by the Health Ministry Screening Committee of India.

## Results

Overall, 1004 participants were enrolled between April 2022 and August 2023 and included in this analysis. The median age was 27 years (interquartile range 23–36), with almost half (44%) aged 18–25 years. 49% had a college degree or more and 3.1% had education <4th standard (i.e., 4th grade); 55% were employed full-time, and 29% reported an income of < INR 10,000/month (< USD ~$113/month). Almost a similar proportion of participants were aware of pre-exposure prophylaxis (PrEP) (31%) and post-exposure prophylaxis (PEP) (27%).

Only 291 participants (29%) were aware of U = U. In the bivariate analysis (shown in Table 1), MSM who were aware of U = U were more likely to have a monthly income above INR 20,000 (USD 226) compared to those with an income less than or equal to INR 10,000 (USD 113) (35% vs. 25%; *P* < 0.01). Awareness was also higher among those who took the survey in English than in Hindi (34% vs. 19%; *P* < 0.001). Participants who had ever tested for HIV showed greater awareness of U = U (38%) than those who had never been tested for HIV (24%), *P* < 0.001. Similarly, those reporting easy access to HIV testing were more likely to be aware of U = U (33%) than those with difficult access (24%), *P* < 0.01. Participants who were aware of HIV prevention methods such as PrEP (46%) and PEP (50%) were significantly more likely to be aware of U = U, compared to those unaware of PrEP (21%) and PEP (21%), *P* < 0.001.

**Table 1:** Participant characteristics associated with awareness of Undetectable=Untransmittable and HIV treatment optimism among men who have sex with men in Mumbai (*N* = 1004)

Variable	Overall *N* (%)	Unaware (U = U) *N* (%)	Aware (U = U) *N* (%)	χ² value	*p*-value^e^	U = U Awareness; Adjusted Prevalence Ratio, aPR (95% CI)	HIV treatment Optimism; Adjusted Prevalence Ratio, aPR (95% CI)
*Total*	1004	713 (71%)	291 (29%)				
*Age* ^f^, *Mean* *(±* ***SD*)**	28 (± 7)	28 (± 7)	28 (± 7)	-0.03	0.974	-	1.04 (1.01, 1.06)^c^
*Age (Categorical)*				0.74	0.900		
18–25	438 (44%)	307 (70%)	131 (30%)				
26–35	424 (42%)	306 (72%)	118 (28%)				
36–45	112 (11%)	80 (71%)	32 (29%)				
46 and above	30 (3%)	20 (67%)	10 (33%)				
*Education*				6.97	0.073		
Less than 4th standard	31 (3.1%)	21 (68%)	10 (32%)				
10th standard	169 (17%)	124 (73%)	45 (27%)				
12th standard	309 (31%)	234 (76%)	75 (24%)				
College graduation or post-graduation	495 (49%)	334 (67%)	161 (33%)				
*Employment*				0.53	0.900		
Student	130 (13%)	90 (69%)	40 (31%)				0.74 (0.47, 1.15
Part-time	154 (15%)	107 (69%)	47 (31%)				0.89 (0.59, 1.32)
Full-time	554 (55%)	397 (72%)	157 (28%)				*Reference*
Retired/Unemployed	166 (17%)	119 (72%	47 (28%)				0.09 (0.00, 1.58)
*Income (n = 1002)*				9.41	**0.009**		
<=10,000	295 (29%)	221 (75%)	74 (25%)				
10,001–20,000	402 (40%)	295 (73%)	107 (27%)				
>20,000	305 (30%)	197 (65%)	108 (35%)				
Missing	2	0	2				
*Language*				22.58	**< 0.001**		
English	678 (68%)	449 (66%)	229 (34%)			*Reference*	*Reference*
Hindi	326 (32%)	264 (81%)	62 (19%)			0.51 (0.36, 0.72)^c^	0.58 (0.42,0.80)^c^
*Ever tested for HIV*				19.69	**< 0.001**		
Yes	348 (35%)	217 (62%)	131 (38%)			1.45 (0.80, 2.65)	
No	586 (58%)	445 (76%)	141 (24%)			0.99 (0.55, 1.77)	
Don’t know / not sure	70 (7%)	51 (73%)	19 (27%)			*Reference*	
*Access to HIV testing*				10.55	**0.009**		
Easy	560 (56%)	374 (67%)	186 (33%)			1.40 (1.03, 1.89)^a^	1.27 (0.96,1.68)
Difficult/Neither	444 (44%)	339 (76%)	105 (24%)			*Reference*	*Reference*
*Perceived HIV risk*				2.59	0.274		
Likely	266 (26%)	181 (68%)	85 (32%)				*Reference*
Neutral	287 (29%)	213 (74%)	74 (26%)				0.67 (0.55, 0.80) ^c^
Not likely	451 (45%)	319 (71%)	132 (29%)				0.93 (0.80, 1.07)
*PrEP awareness*				62.07	**< 0.001**		
Aware	308 (31%)	166 (54%)	142 (46%)			2.23 (1.61, 3.07)^c^	1.98 (1.41,2.77)^c^
Not Aware	696 (69%)	547 (79%)	149 (21%)			*Reference*	*Reference*
*PEP awareness*				82.27	**< 0.001**		
Aware	274 (27%)	136 (50%)	138 (50%)			2.69 (1.62, 4.47)^c^	
Not Aware	730 (73%)	577 (79%)	153 (21%)			*Reference*	
*HIV treatment Optimism*	503 (50%)	317(63%)	186 (37%)	30.52	0.072		
No	501 (50%)	396(79%)	105 (21%)				
*History of Sex work*	160 (16%)	106 (66%)	54 (34%)	10.95	0.150		
*Condom Use*				0.23	0.600		
Always	504 (50%)	354 (70%)	150 (30%)				
Not Always	500 (50%)	359 (72%)	141 (28%)				
*Targeted HIV Prevention Intervention Participation*^**d**^ (Yes)	111 (11%)	60 (54%)	51 (46%)	16.8	**< 0.001**	2.24 (1.40, 3.56)^c^	
No	890 (89%)	652 (73)	238 (27%)			*Reference*	
*Searched for sexual health information (online)*	518 (52%)	328 (63%)	190 (37%)	30.02	**< 0.001**	1.58 (1.16, 2.14)^b^	1.61 (1.22,2.12)^c^
No	486 (48%)	385 (79%)	101 (21%)			*Reference*	*Reference*
*Out to Everyone*	220 (22%)	154 (70%)	66 (30%)	0.09	0.707		1.95 (1.43,2.65)^c^
No	784 (78%)	559 (71%)	225 (29%)				*Reference*
*Someone to talk about sex concerns*				5.95	0.051		
Always	100 (10%)	64 (64%)	36 (36%)				
Sometimes	382 (38%)	262 (74%)	120 (26%)				
Never/Rarely	522 (52%)	387 (69%)	135 (31%				
*Sexual Orientation*				0.43	0.822		
Bisexual	293 (29%)	211 (72%)	82 (28%)				*Reference*
Gay/Double-Decker/Kothi/Panthi	644 (64%)	453 (70%)	191 (30%)				0.49 (0.23,1.02)
Heterosexual/Other	67 (7%)	49 (73%)	18 (27%)				1.09 (0.79,1.50)
*Source of enrolment*				15.69			
Dating Apps	425 (42%)	324 (76%)	101 (24%)		**0.003**		
Messaging Apps	332 (33%)	228 (69%)	104 (31%)				
Snowball	19 (2%)	13 (68%)	6 (32%)				
Social media	182 (18%)	112 (62%)	70 (38%)				
Source Not Captured	46 (5%)	36 (78%)	10 (22%)				

^a^ *p* < 0.05

^b^ *p* < 0.01

^c^ *p* < 0.001

^d^ Targeted HIV Prevention Intervention (TI) are existing in-person prevention interventions in India

^e^ Bolded p values are statistically significant at *p* < 0.05

^f^ Welch two-sample *t*-test

In multivariable analysis (Table 1), participants completing the survey in Hindi versus English had lower awareness of U = U (aPR = 0.51 [95% CI 0.36, 0.72], *P* < 0.001). Increased awareness of U = U was associated with being aware of biomedical prevention methods, such as PrEP (aPR = 2.23 [1.61, 3.07]; *P* < 0.001) and PEP (aPR = 2.69 [1.62, 4.47]; *P* < 0.001), and searching online for sexual health information (aPR = 1.58 [1.16, 2.14]; *P* < 0.01). Awareness of U = U was also higher among participants who were part of an existing MSM-focused HIV prevention program (aPR = 2.24 [1.40, 3.56]; *P* < 0.001. There was no significant association between U = U awareness and HIV testing history. Table 1 reports the adjusted prevalence ratios and 95% confidence intervals for each variable included in the final model.

With regard to HIV treatment optimism, only about half of the participants (*n* = 503, 50%) agreed that HIV treatment could lead to a healthy and long life. In multivariable analysis (Table 1), participants who were aware of PrEP (aPR = 1.98 [1.41, 2.77]; *P* < 0.001) and used online platforms to search for sexual health information were more likely to have HIV treatment optimism (aPR = 1.61 [1.22, 2.12]; *P* < 0.001). Furthermore, participants who were open about their sexual orientation to everyone (aPR = 1.95 [1.43, 2.65]; *P* < 0.001) had HIV treatment optimism. Participants who reported being neutral (response option) about their HIV risk perception had lower HIV treatment optimism (aPR = 0.67 [0.55, 0.80]; *P* < 0.001). The adjusted prevalence ratios and 95% confidence intervals for each variable included in the final model are shown in Table 1. The bivariate analysis is provided in Supplementary Table 1.

## Discussion

Our findings revealed low U = U awareness and limited HIV treatment optimism among MSM in India, particularly among those with no or infrequent recent HIV testing. Fewer than a third were aware of U = U, while only half the participants expressed treatment optimism. Notably, certain groups, such as Hindi survey respondents, had even lower levels of awareness and treatment optimism. These low awareness and optimism levels contrast sharply with studies from higher-income countries. For example, an online survey of MSM in the U.S. found 85% awareness of U = U, although less than half (42.5%) trusted this fact [9]. Similarly, a comparative study across Latin American countries reported high U = U awareness in Mexico (89.2%), Brazil (88.6%), and Peru (63.7%) [6]. In comparison, the only other Indian study of U = U awareness found just 14% awareness [7], possibly due to sampling differences. Our sample was drawn from the Mumbai metropolitan area, whereas the prior study surveyed participants across India. Given Mumbai’s likely higher penetration of HIV-related outreach compared to smaller cities, differences in awareness levels may be expected. Additional factors such as survey timing (i.e., prior study enrolled in 2022 versus ours from 2021 to 2023), language preference (English vs. Hindi), income, and educational status may also have contributed to these observed differences. These disparities highlight the urgent need for focused educational and awareness campaigns in India, particularly among underserved MSM, as U = U knowledge and treatment optimism could lower stigma and improve HIV testing, prevention, and treatment engagement [4].

Several factors may account for the observed lack of awareness and treatment optimism. India’s Test and Treat policy facilitates immediate antiretroviral treatment for anyone diagnosed with HIV, enabling them to rapidly achieve an undetectable viral load [2]. Consequently, people living with HIV may be informed about U = U, whereas this information is not systematically disseminated to individuals who do not have HIV or are unaware of their status [10]. As our study enrolled MSM without HIV, this difference may contribute to their lack of awareness of U = U. Furthermore, there have been no widespread public health campaigns promoting U = U in India, either online or offline. The stigma associated with HIV and MSM may also hinder discussions and spread of accurate information about U = U and HIV treatment benefits. Lastly, HIV prevention efforts in India, as in many other regions globally, have usually prioritized and promoted condoms for all MSM, but information about HIV viral suppression has been specifically targeted to people living with HIV, resulting in differential messaging [11].

Our study found that awareness of biomedical HIV prevention tools, such as PrEP and PEP, searching for sexual health information online, and completing the survey in English, were associated with awareness of U = U and treatment optimism. These findings align with results from a web-based survey among sexual and gender minority individuals in Brazil and the United States, where PrEP awareness was associated with higher awareness and perceived accuracy of U = U [9, 12]. Together, these findings suggest that individuals knowledgeable about newer HIV prevention methods or those who seek sexual health information online are more likely to encounter HIV-related advancements including U = U, as this information is likely mostly available in English online. Our findings underscore the importance of online platforms as key resources for sexual health information and the need for dissemination of U = U information in local languages for a broader reach.

In this study, participants engaged in MSM-focused HIV prevention programs – known as Targeted Interventions (Tis) in India – were more likely to be aware of U = U. These programs link MSM to Integrated Counselling and Testing Centres and provide a non-discriminatory setting to access HIV testing and status-neutral linkage to care [11]. The TIs also conduct workshops that may include information about biomedical prevention (i.e., PrEP and PEP) and potentially U = U messaging. However, the extent of dissemination of this information within these programs remains unknown and likely limited to MSM registered with the TIs. To our knowledge, there has been no nationwide U = U campaign in India. Nonetheless, TIs can offer stigma-free spaces for sexual health discussions and may therefore represent a strategic platform to expand U = U awareness among Indian MSM.

This study is not without limitations. First, we utilized a convenience sample of MSM on diverse dating apps and social media platforms and used self-administered surveys, which may not represent all Indian MSM in Mumbai (e.g., precluding those with low literacy). Second, given the cross-sectional design, we cannot infer causality. Additionally, the study depended on self-reported data, which may be subject to social desirability or recall bias. Despite these limitations, this study sheds light on a diverse and often unreached sample of MSM, providing insights on an understudied topic in India.

## Conclusion

Our findings revealed low levels of U = U awareness and treatment optimism among MSM in India, with certain groups having even greater informational gaps. These disparities underscore the need for widespread dissemination of accurate messaging of U = U and HIV treatment benefits among diverse Indian MSM. Such dissemination efforts could significantly support public health goals of reducing stigma and enhancing HIV testing, prevention, and treatment engagement. Integrating U = U education into the existing public health infrastructure and online platforms may offer scalable and sustainable pathways to advance HIV prevention and merit further implementation research.

## Supplementary Information

Below is the link to the electronic supplementary material.

Supplementary Material 1

## Data Availability

The dataset can be made available after publication of primary findings from the corresponding author upon reasonable request, with appropriate ethical approvals and data use agreements.
