# Supplementary material for: Undetectable = Untransmittable (U = U) Awareness and HIV Treatment Optimism Among Men Who Have Sex with Men in Mumbai, India
Source: AIDS Behav. 2026 Feb 24;30(8):2394–401. doi: 10.1007/s10461-026-05063-z (PMC13486109; doi:10.1007/s10461-026-05063-z)
Supplement: Supplementary file 1 [file 10461_2026_5063_MOESM1_ESM.docx]

**Title: Undetectable = Untransmittable (U=U) Awareness and HIV Treatment Optimism Among Men Who Have Sex with Men in Mumbai and Thane, India**

**Supplementary Table 1. Correlates of HIV treatment optimism among men who have sex with men in Mumbai and Thane, India**

| **Variable** | **Overall**  **N (%)** | **Agree**  **n (%)** | **Disagree**  **n (%)** | **χ² value** | **p-value**^b^ |
| --- | --- | --- | --- | --- | --- |
| **Total** | 1004 | 503 (50%) | 501 (50%) |  |  |
| **Age**^c^**, *Mean* (± *SD*)** | 28 (±7) | 29 (±8) | 27 (±7) | 3.60 | **<0.001** |
| **Age (Categorical)** |  |  |  | 14.98 | **0.002** |
| 18-25 | 438 (44%) | 195 (44%) | 243 (56%) |  |  |
| 26-35 | 424 (42%) | 220 (52%) | 204 (48%) |  |  |
| 36-45 | 112 (11%) | 67 (60%) | 45 (40%) |  |  |
| 46+ | 30 (3%) | 21 (70%) | 9 (30%) |  |  |
| **Education** |  |  |  | 22.23 | **<0.001** |
| Less than 4^th^ standard | 31 (3.1%) | 10 (32%) | 21 (68%) |  |  |
| 10^th^ standard | 169 (17%) | 73 (43%) | 96 (57%) |  |  |
| 12^th^ standard | 309 (31%) | 136 (44%) | 173 (56%) |  |  |
| Graduation/post- graduation | 495 (49%) | 284 (57%) | 211 (43%) |  |  |
| **Employment** |  |  |  | 2.35 | 0.500 |
| Student | 130 (13%) | 57 (44%) | 73 (56%) |  |  |
| Part-time | 154 (15%) | 78 (51%) | 76 (49%) |  |  |
| Full-time | 554 (55%) | 283 (51%) | 271 (49%) |  |  |
| Retired/Unemployed | 166 (17%) | 85 (51%) | 81 (49%) |  |  |
| **Income (n=1002)** |  |  |  | 30.27 | **<0.001** |
| <=10,000 | 295 (29%) | 123 (42%) | 172 (58%) |  |  |
| 10,001 – 20,000 | 402 (40%) | 188 (47%) | 214 (53%) |  |  |
| >20,000 | 305 (30%) | 192 (63%) | 113 (37%) |  |  |
| Missing | 2 |  | 2 |  |  |
| **Language** |  |  |  | 20.79 | **<0.001** |
| English | 678 (68%) | 374 (55%) | 304 (45%) |  |  |
| Hindi | 326 (32%) | 129 (40%) | 197 (60%) |  |  |
| **Ever tested for HIV** |  |  |  | 18.99 | **<0.001** |
| Yes | 348 (35%) | 205 (59%) | 143 (41%) |  |  |
| No | 586 (58%) | 260 (44%) | 326 (56%) |  |  |
| Don’t know/ not sure | 70 (7%) | 38 (54%) | 32 (46%) |  |  |
| **Access to HIV testing** |  |  |  | 9.26 | **0.002** |
| Easy | 560 (56%) | 305 (54%) | 255 (46%) |  |  |
| Difficult/Neither | 444 (44%) | 198 (45%) | 246 (55%) |  |  |
| **Perceived HIV risk** |  |  |  | 31.59 | **<0.001** |
| Likely | 266 (26%) | 138 (52%) | 128 (48%) |  |  |
| Neutral | 287 (29%) | 105 (37%) | 182 (63%) |  |  |
| Not likely | 451 (45%) | 260 (58%) | 191 (42%) |  |  |
| **PrEP awareness** |  |  |  | 34.95 | **<0.001** |
| Aware | 308 (31%) | 198 (64%) | 110 (36%) |  |  |
| Not Aware | 696 (69%) | 305 (44%) | 391 (56%) |  |  |
| **PEP awareness** |  |  |  | 23.52 | **<0.001** |
| Aware | 274 (27%) | 172 (63%) | 102 (37%) |  |  |
| Not Aware | 730 (73%) | 331 (45%) | 399 (55%) |  |  |
| **History of Sex work** | 160 (16%) | 67 (42%) | 93 (58%) | 4.77 | **0.023** |
| No | 844 (84%) | 436 (52%) | 408 (48%) |  |  |
| **Condom Use** |  |  |  | 12.49 | **<0.001** |
| Always | 504 (50%) | 281 (56%) | 223 (44%) |  |  |
| Not Always | 500 (50%) | 222 (44%) | 278 (56%) |  |  |
| **Targeted HIV Prevention Intervention Participation^a^** | 111 (11%) | 41 (37%) | 70 (63%) | 8.13 | **0.003** |
| No | 890 (89%) | 461 (52%) | 429 (48%) |  |  |
| **Someone to talk about sex concerns** |  |  |  | 26.27 | **<0.001** |
| Always | 100 (10%) | 71 (71%) | 29 (29%) |  |  |
| Sometimes | 382 (38%) | 202 (53%) | 180 (47%) |  |  |
| Never/Rarely | 522 (52%) | 230 (44%) | 292 (60%) |  |  |
| **Searched for sexual health information online** | 518 (52%) | 304 (59%) | 214 (41%) | 30.86 | **<0.001** |
| No | 486 (48%) | 199 (41%) | 287 (59%) |  |  |
| **Out to everyone** | 220 (22%) | 122 (55.5) | 98 (44.5) | 2.96 | 0.072 |
| No | 784 (78%) | 381 (48.6) | 403 (51.4) |  |  |
| **Sexual Orientation** |  |  |  | 1.34 | 0.085 |
| Bisexual | 293 (29%) | 148 (51%) | 145 (49%) |  |  |
| Gay/Double-Decker/Kothi/Panthi | 644 (64%) | 326 (51%) | 318 (49%) |  |  |
| Heterosexual/Other | 67 (7%) | 29 (43%) | 38 (57%) |  |  |
| **Source of enrolment** |  |  |  | 11.98 | **0.017** |
| Dating Apps | 425 (42%) | 197 (46%) | 228 (54%) |  |  |
| Messaging Apps | 332 (33%) | 165 (50%) | 167 (50%) |  |  |
| Snowball | 19 (2%) | 13 (68%) | 6 (32%) |  |  |
| Social media | 182 (18%) | 108 (59%) | 74 (41%) |  |  |
| Source Not Captured | 46 (5%) | 20 (44%) | 26 (56%) |  |  |

^a^ Targeted HIV Prevention Intervention (TI) are existing in-person prevention interventions in India

^b^ Bolded p values are statistically significant at p<0.05

^c^ Welch two-sample *t*-test

**Supplementary Figure 1. Variable Selection Process Using LASSO Poisson Regression**
